# Differentiation of Human Adipose Derived Stem Cells into Smooth Muscle Cells Is Modulated by CaMKII*γ*

**DOI:** 10.1155/2016/1267480

**Published:** 2016-07-14

**Authors:** Kaisaier Aji, Munila Maimaijiang, Abudusaimi Aimaiti, Mulati Rexiati, Baihetiya Azhati, Hamulati Tusong, Lei Cui

**Affiliations:** ^1^Department of Urology, The First Affiliated Hospital of Xinjiang Medical University, Urumqi, Xinjiang 830054, China; ^2^Prevention and Health Care Department, The Second Affiliated Hospital of Xinjiang Medical University, Urumqi, Xinjiang 830063, China; ^3^Department of Joint Surgery, The First Affiliated Hospital of Xinjiang Medical University, Urumqi, Xinjiang 830054, China; ^4^Institute of Medical Science, Beijing Shijitan Hospital, Capital Medical University, Beijing 100038, China

## Abstract

The multifunctional Ca^2+^/calmodulin-dependent protein kinase II (CaMKII) is known to participate in maintenance and switches of smooth muscle cell (SMC) phenotypes. However, which isoform of CaMKII is involved in differentiation of adult mesenchymal stem cells into contractile SMCs remains unclear. In the present study, we detected *γ* isoform of CaMKII in differentiation of human adipose derived stem cells (hASCs) into SMCs that resulted from treatment with TGF-*β*1 and BMP4 in combination for 7 days. The results showed that CaMKII*γ* increased gradually during differentiation of hASCs as determined by real-time PCR and western blot analysis. The siRNA-mediated knockdown of CaMKII*γ* decreased the protein levels and transcriptional levels of smooth muscle contractile markers (a-SMA, SM22a, calponin, and SM-MHC), while CaMKII*γ* overexpression increases the transcriptional and protein levels of smooth muscle contractile markers. These results suggested that *γ* isoform of CaMKII plays a significant role in smooth muscle differentiation of hASCs.

## 1. Introduction

Adipose derived stem cell (ASCs) is self-renewing multipotent cells that have significant clinical potential in cellular therapy for tissue regeneration [1]. ASCs can be induced to differentiate along multiple lineages, including osteocytes [2], neural cells [3], and muscular cells [4]. Differentiation of ASCs into blood vessel smooth muscle cells (SMCs) under stimulation of transforming growth factor-*β*1 (TGF-*β*1) and bone morphogenetic protein-4 (BMP4) has been fulfilled in our previous study [5]. The differentiated ASCs acquired SMC phenotype as evidenced by their expression of specific structural proteins and their contractility in response to contractile agonist. Furthermore, an elastic blood vessel wall was engineered under pulsatile conditions using smooth muscle cells differentiated from ASCs and polyglycolic acid scaffold, showing that hASCs can serve as an alternative cell source for SMCs in blood vessel engineering [6]. However, the mechanism underlying differentiation of ASCs into smooth muscle lineage remains unclear.

Homeostasis of intracellular Ca^2+^ maintains proliferation, extracellular matrix production, and phenotypic switch of differentiated SMCs. As a critical mediator of Ca^2+^ signals, the multifunctional serine/threonine Ca^2+^/calmodulin-dependent protein kinase II (CaMKII), which consists of 4 different isoforms with distinct expression patterns [7, 8], plays a critical role in modulating physiology and pathological process of SMCs [9, 10]. Among the four homologous CaMKII isoforms (*α*, *β*, *δ*, and *γ*), CaMKII isoform *δ* has been generally accepted as a major regulator in promoting SMCs synthetic phenotype functions [11, 12]. Recently, CaMKII*γ* isoforms were shown to associate with acquirement of contractile activity of SMCs. Antisense knockdown of CaMKII*γ* inhibited extracellular signal-related kinase (ERK) activation, myosin phosphorylation, and contractile force in differentiated SMCs [13]. These results indicated that *γ* isoform of CaMKII regulates smooth muscle differentiation. But there are no reports about whether CaMKII*γ* regulates smooth muscle differentiation of ASCs.

In the present study, we hypothesized that *γ* isoform of CaMKII participates in smooth muscle differentiation of ASCs. We found that, in parallel to differentiation of ASCs, expression of CaMKII*γ* was significantly upregulated. Inhibition of CaMKII*γ* with si-RNA decreased smooth muscle differentiation of ASCs. This result indicted that, in addition to modulating phenotype switch of mature differentiated SMCs, CaMKII*γ* showed fundamental function in regulation of smooth muscle differentiation of adult mesenchymal stem cells.

## 2. Materials and Methods

### 2.1. Cell Culture and Smooth Muscle Differentiation

Human adipose derived stem cells isolated from fresh human lipoaspirates (the process was approved by the Research Ethical Committee of the First Affiliated Hospital of Xinjiang Medical University), cultured in growth medium, comprised LG-DMEM (Gibco) supplemented with 10% FBS, 100 U/mL penicillin (Sigma-Aldrich), and 100 mg/mL streptomycin (Sigma-Aldrich) as previously described [5]. Cells from passages 3 to 5 were used in the following study. Smooth muscle cell differentiation of hASCs was induced using differentiation medium containing 1% FBS, 5 ng/mL TGF-*β*1, and 2.5 ng/mL BMP4. Smooth muscle differentiation of hASCs was evaluated by quantitative real-time PCR (qRT-PCR) and immunofluorescent staining.

### 2.2. Quantitative Real-Time Reverse Transcription PCR

RNA was extracted with TRIzol reagent (Invitrogen, Carlsbad, CA) and PrimeScript*™* RT Master Mix (Takara, Japan) was used for cDNA synthesis and the reactions were performed in a T3 thermocycler (Biometra). qRT-PCR was performed by using a 7500 Fast Real-Time PCR system (Applied Biosystems, USA) according to the manufacturer's protocol. Primers used in the PCR reactions were listed in Table 1. SYBER Green Premix Ex Taq (Takara, Japan) was used in each reaction. The relative expression of mRNA was normalized to *β*-actin. Fold change was calculated by using the ΔΔCt method of relative quantification. All experiments were repeated in triplicate.

### 2.3. Western Blotting

Western blot test was used to identify expression of SMC proteins. Cells were lysed in RIPA buffer with protease and phenylmethylsulfonyl fluoride (PMSF; Roche, Indianapolis, IN, USA). The protein concentration was assayed using the BCA method (Bio-Rad). Approximately 20 to 50 *μ*g of total protein samples was loaded on a 10% SDS-PAGE after denaturation by boiling for 10 min. The separated proteins transferred to polyvinylidene difluoride membranes. Membranes were blocked by incubation in Tris-buffered saline containing 0.05% Tween 20 and 5% skimmed milk with constant shaking for 1 h. The membrane was probed with antibodies against a-SMA (1 : 1000, Abcam, Cambridge, UK), calponin (1 : 1000, Abcam, Cambridge, UK), SM22a (1 : 1000, Abcam, Cambridge, UK), SM-MHC (1 : 1000, Abcam, Cambridge, UK), smoothelin-like 2 (1 : 2000, Santa Cruz, Dallas, USA), ACLP (1 : 1000, Abcam, Cambridge, UK), CaMKII*α* (1 : 5000, Abcam, Cambridge, UK), CaMKII*β* (1 : 1000, Abcam, Cambridge, UK), CaMKII*γ* (1 : 500, Abcam, Cambridge, UK), and CaMKII*δ* (1 : 1000, Santa Cruz, Dallas, USA) overnight at 4°C. GAPDH was used as an internal loading control. The membranes were washed three times with TBST and incubated with the appropriate secondary antibodies at room temperature for 1 h and detected using enhanced chemiluminescence.

### 2.4. Overexpression and Knockdown of CaMKII*γ*

For the overexpression analysis, CaMKII*γ* adenovirus vectors were constructed by using the AdEasy system (Agilent Technologies, Santa Clara, CA, USA). CaMKII*γ* was cloned from pRC/CMV plasmid with the forward primer 5′-GTCTGTCAACGATCCACGGT-3′ and the reverse primer 5′-TCTGCCTGCCAACTGAGAAG-3′. The empty vector served as the green fluorescent protein (GFP) control. hASCs cell passages 3–5 were incubated with adenoviruses expressing GFP and CaMKII*γ* at an MOI of 250 for 48 h [14]. CaMKII*γ* mRNA and protein expressions were analyzed by qRT-PCR and western blotting. For knockdown of CaMKII*γ*, siRNAs were synthesized by Usen Biotech Co. (Shanghai, China). The sequence of the CaMKII*γ* siRNA wa: 5′-TACGATACAAGGCTGTTAGAGAG-3′. A random sequence was used as the negative control (NC). hASCs were seeded into 6-well plates and transfected with siRNA at 100 pmol/well using Lipofectamine 2000 (Invitrogen Corp.), according to the manufacturer's instructions. The medium was replaced 6 h later and the cells were collected 48 h after transfection for total RNA isolation and protein harvesting. Transfection efficiency was evaluated by qRT-PCR and western blotting.

### 2.5. Immunofluorescent Staining

Cells were fixed with paraformaldehyde and incubated with the following primary antibodies: rabbit polyclonal anti-a-SMA, rabbit polyclonal anti-SM22a, and rabbit polyclonal anti-SM-MHC antibodies for 60 min at room temperature, and then they were washed with PBS for three times. Alexa Fluor 594-conjugated donkey anti-rabbit secondary antibody (R37119; Thermo Fisher, US) was used to detect the localization of anti-a-SMA, anti-SM22a, and anti-SM-MHC antibodies, respectively. Nuclear staining was done with 4′,6-diamidino-2-phenylindole (DAPI). The images were viewed by a confocal laser scanning microscope (TCS, SP8; Leica, Germany).

### 2.6. Statistical Analysis

The results presented are average of at least three experiments each performed in triplicate with mean ± SD. Statistical analysis was performed using Student's *t*-test. *P* < 0.05 was considered statistically significant.

## 3. Results

### 3.1. Smooth Muscle Differentiation of hASCs

**Figure 1:**
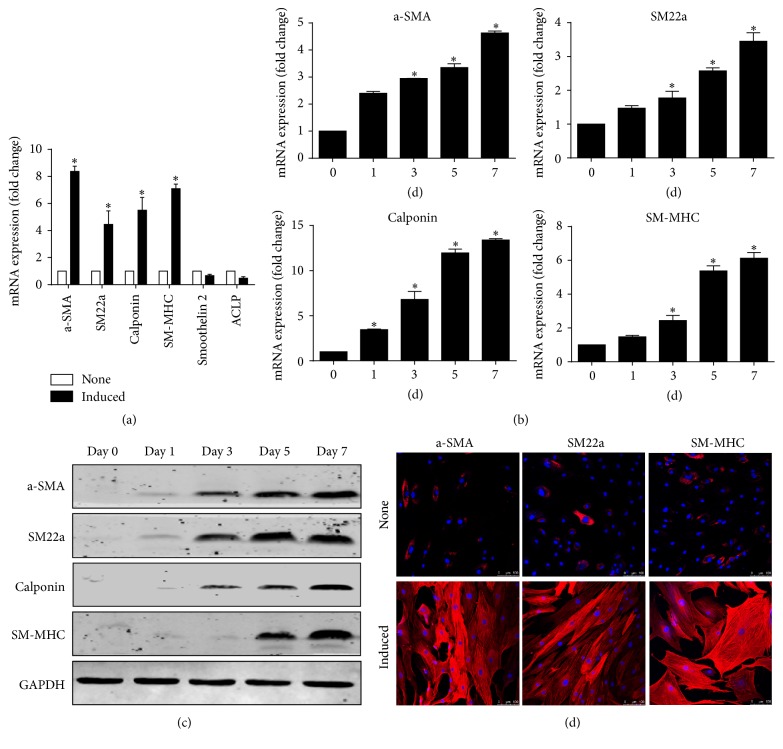
Human ASCs were induced to differentiate into SMCs by treatment with TGF-*β*1 and BMP4 for 7 days. (a) Quantitative real-time PCR experiments were carried out to measure the expression level of smooth muscle specific markers. (b, c) Time course of the expression of SMC differentiation markers detected by qRT-PCR and western blot analysis, respectively. (d) Immunofluorescent staining of smooth muscle specific markers (red), respectively. Nuclear were counterstained with DAPI (blue). Scale bars: 100 *μ*m. Data represent means ± SE, *n* = 3 (^*∗*^ *P* < 0.05 versus controls).

As determined by qRT-PCR, expression of smooth muscle specific contractile genes including *α*-SMA, SM22a, calponin, and SM-MHC was detected with significant increase at 7 days in hASCs stimulated with combination of TGF-*β*1 and BMP4. But expressions of CArG-independent smooth muscle differentiation markers like smoothelin-like 2 and ACLP (also known as AE binding protein 1) were not different from noninduced group (Figures 1(a), 1(b), and 1(c)). To ascertain the results of qRT-PCR, expression of intracellular *α*-SMA, SM22a, and SM-MHC was analyzed by immunofluorescent staining. As shown in Figure 1(d), expressions of these markers were remarkably enhanced in hASCs stimulated with the combination of TGF-*β*1 and BMP4 for 7 days.

### 3.2. CaMKII*γ* Was Highly Expressed during Smooth Muscle Differentiation

**Figure 2:**
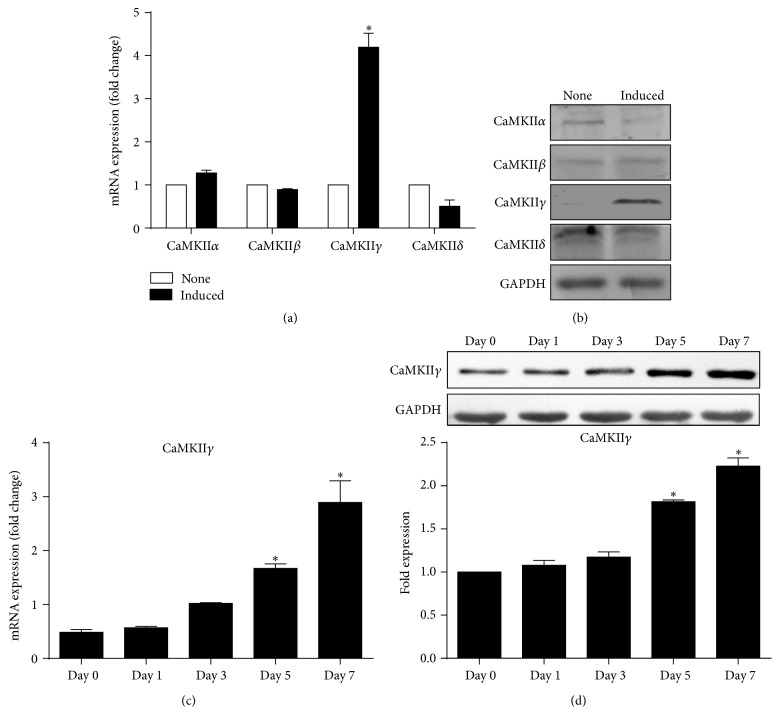
Expression of CaMKII isoforms in hASCs subjected to combined treatment with TGF-*β*1 and BMP4 for 7 days. mRNA (a) and protein (b) expressions of CaMKII isoforms after smooth muscle differentiation of hASCs. (c) qRT-PCR analysis of CaMKII*γ* expression at 1, 3, 5, and 7 days, respectively. (d) Expression of CaMKII*γ* detected by western blot at 1, 3, 5, and 7 days of stimulation. ^*∗*^ *P* < 0.05 when (a) versus none, (b) versus day 0, and (d) versus day 0.

We examine expression of four isoforms of CaMKII on differentiation of hASCs into smooth muscle cells. As shown in Figures 2(a) and 2(b), among the four isoforms of the CaMKII, only the *γ* isoform has a significant change compared with the noninduced group. We also detected expressions of CaMKII*γ* gene at 1, 3, 5, and 7 days after induction by qRT-PCR analysis. We found that expression of CaMKII*γ* began to increase after 3 days and reached significant high level at 5 and 7 days, respectively (Figure 2(c)). By western blot analysis, it was found that, during treatment by TGF-*β*1 and BMP4 in combination, protein levels of CaMKII*γ* gradually increased after 3 days in induced hASCs (Figure 2(d)).

### 3.3. CaMKII*γ* Has Positive Effect on Smooth Muscle Differentiation of hASCs

**Figure 3:**
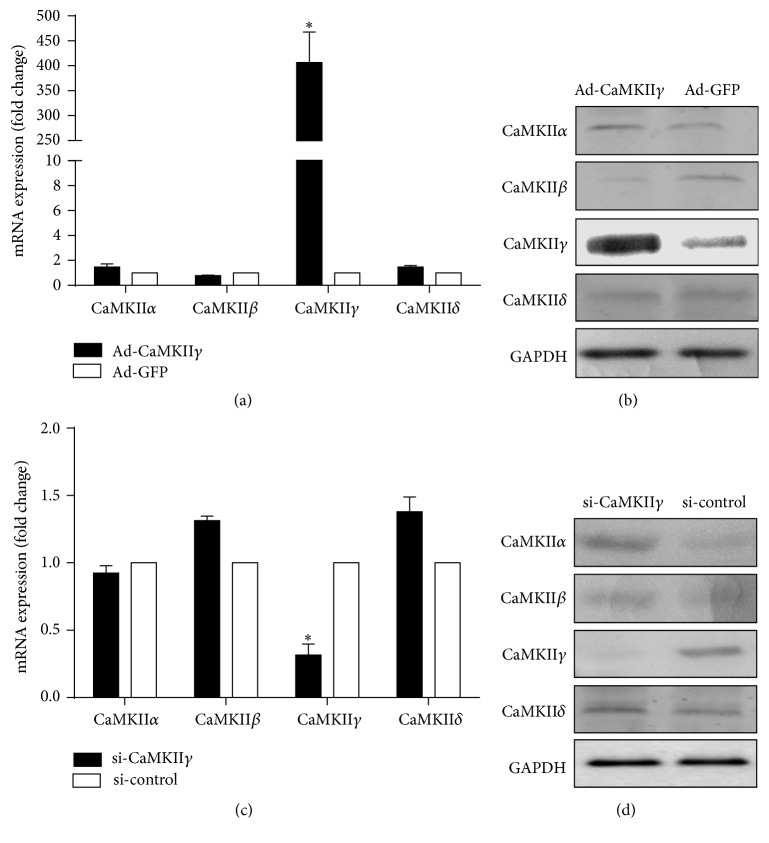
Cell transfection efficiency. CaMKII mRNA (a) and protein (b) expression levels in hASCs transfected with CaMKII*γ* and control. (c) qRT-PCR analysis of CaMKII*α*, CaMKII*β*, CaMKII*γ*, and CaMKII*δ* in hASCs transfected with siRNA targeting CaMKII*γ* (si-CaMKII*γ*) and nontargeting siRNA (si-control) for 48 hours, respectively. (d) Detection of CaMKII isoforms by western blot in hASCs transfected with siRNA targeting CaMKII*γ* (si-CaMKII*γ*) and nontargeting siRNA (si-control). ^*∗*^ *P* < 0.05 when (a) versus ad-GFP and (c) si-control.

To further understand the role of CaMKII*γ* in the control of contractile gene expression, we constructed overexpression vector of CaMKII*γ* and transfected hASCs. Figures 3(a) and 3(b) show that CaMKII*γ* overexpressed in the transfected cell and ad-CaMKII*γ* is specific to *γ* isoform of CaMKII only. For knockdown of CaMKII*γ*, siRNA against CaMKII*γ* or si-control transfected into hASCs for 48 hours. The qRT-PCR results showed that mRNA levels of CaMKII*γ*, but not other isoforms of CaMKII, were downregulated by si-CaMKII*γ* more than 70% compared to si-control (Figure 3(c)). In addition, CaMKII*γ* protein level decreased by 90% with si-CaMKII*γ* transfection as determined by western blot analysis (Figure 3(d)). These results indicated that transfection of siRNA effectively suppressed expression of CaMKII*γ* and si-CaMKII*γ* is specific to *γ* isoform of CaMKII only.

**Figure 4:**
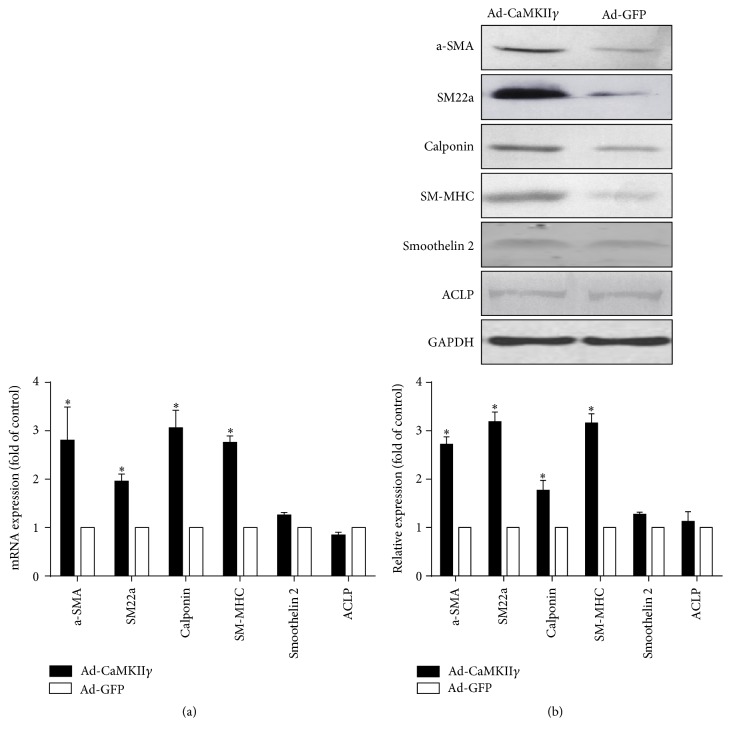
Transfection of CaMKII*γ* upregulated expression of smooth muscle contractile markers. (a) qRT-PCR analysis of smooth muscle specific markers. (b) Western blot analysis of smooth muscle contractile proteins in CaMKII*γ* and control group, respectively. Data represent means ± SE, *n* = 3 (^*∗*^ *P* < 0.05 versus si-control).

We also detected expression levels of smooth muscle contractile markers after transfection of CaMKII*γ*. mRNA expression levels of CArG-dependent smooth muscle differentiation markers was upregulated in the CaMKII*γ* group compared to control group analyzed by qRT-PCR (Figure 4(a)). Western blot analysis also confirms these kinds of expressions (Figure 4(b)). But CArG-independent smooth muscle differentiation markers (smoothelin-like 2 and ACLP) were not changed by CaMKII*γ*.

**Figure 5:**
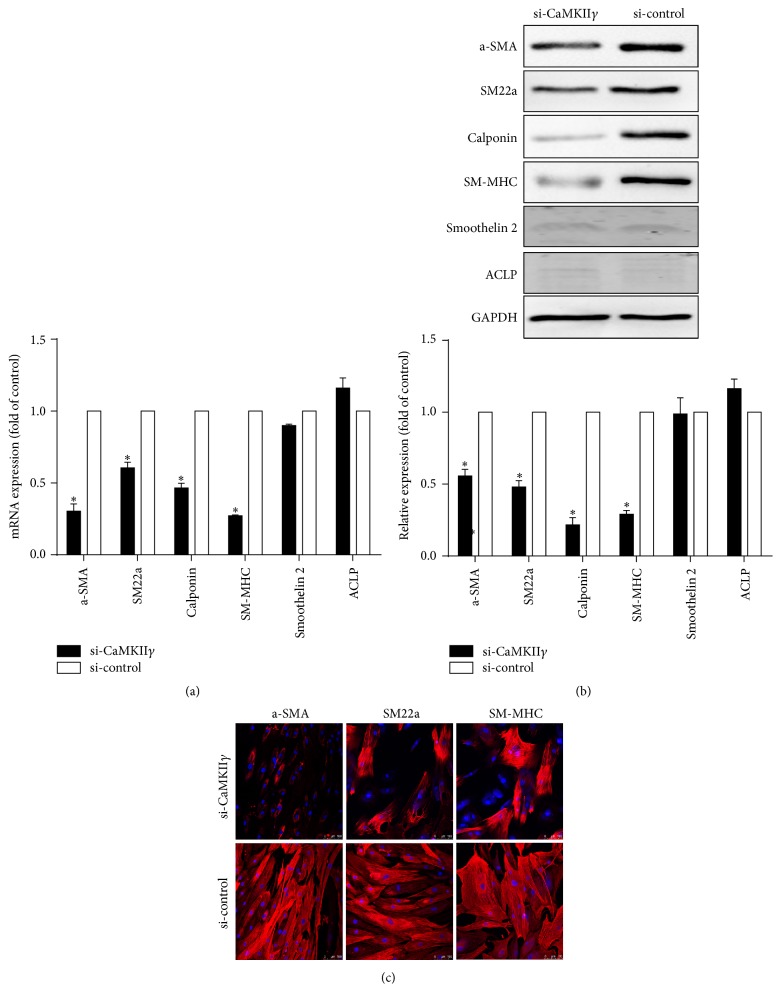
Expression of smooth muscle contractile markers was downregulated in induced hASCs transfected with siRNA against CaMKII*γ*. (a) Expression of smooth muscle contractile markers in si-CaMKII*γ* and si-control group determined by qRT-PCR. Data represent means ± SE, *n* = 3 (^*∗*^ *P* < 0.05 versus si-control). (b) Western blot analysis of smooth muscle contractile proteins in si-CaMKII*γ* and si-control group, respectively. Data represent means ± SE, *n* = 3 (^*∗*^ *P* < 0.05 versus si-control). (c) Immunofluorescent staining of a-SMA, SM22a, and SM-MHC (red) in si-CaMKII and si-control group, respectively. Nuclear were stained with DAPI (blue). Scale bars: 100 *μ*m.

After being transfected with si-CaMKII*γ*, hASCs were induced with exposure to TGF-*β*1 and BMP4 in combination for seven days to differentiate into smooth muscle cells. Compared with si-control group, mRNA expressions of a-SMA, SM22a, calponin, and SM-MHC, but not smoothelin-like 2 and ACLP, in induced hASCs were significantly reduced by transfection of si-CaMKII*γ*, among which decrease of a-SMA and SM-MHC was more evident (Figure 5(a)). As determined by western blot analysis, it was shown that CArG-dependent smooth muscle contractile proteins were downregulated by si-CaMKII*γ* (Figure 5(b)). Furthermore, immunofluorescent staining exhibited reduced distribution of *α*-SMA, SM22a, and SM-MHC in si-CaMKII*γ* transfected hASCs (Figure 5(c)).

## 4. Discussion

Smooth muscle cells (SMCs) play an important role in angiogenesis and regulate blood pressure by contracting and relaxing in response to a variety of stimulus [15]. Thus, any defect or damage in smooth muscle tissue may result in severe dysfunctions of cardiovascular system. Up to now, a variety of cell sources including mature SMCs and adult mesenchymal stem cells have been used in blood vessel tissue engineering to repair vascular defects. Because of their limited ability to proliferate and usually loss of their contractile phenotype in expansion, mature differentiated SMCs have great limitation in tissue engineering. Adipose derived stem cells (ASCs) have advantages in that they are easy to be harvested, have relatively lower donor site morbidity, and can expand more rapidly in vitro [16]. ASCs have the potential to differentiate into functional smooth muscle cells and, therefore, adipose tissue can be a useful source of cells for treatment of injured tissues where smooth muscle plays an important role [17]. Thus, ASCs could be a preferred novel cell source for blood vessel engineering. A better understanding of the molecular mechanisms involved in regulating SMC differentiation is critical for facilitating the development of blood vessel tissue engineering. In previous studies, several transcriptional factors have been reported to be involved in differentiation of ASCs along smooth muscle cell pathway, which includes serum response factor (SRF), myocardin (Myocd), Myocd-related transcription factors (MRTFs) [18–20], and Krüppel-like factor-4 (KLF-4) [21].

CaMKII is a multimeric enzyme and its activity is regulated by the binding of Ca^2+^/calmodulin (CaM), which activates its protein kinase activity and promotes intrasubunit autophosphorylation. The autophosphorylated enzyme retains its kinase activity after the release of Ca^2+^/CaM, a phenomenon called autonomous activity [22]. Although CaMKII is multifunctional and all isoforms appear to have similar peptide substrate specificities and kinetics [23], it remains possible that specific isoforms may be discriminated at the level of protein substrate specificity. Among the four different isoforms of CaMKII, the *α* and *β* isoforms are predominantly expressed in neural system [24] and involved in synaptic plasticity, memory, and learning process [25]. In vitro, signaling through CaMKII*δ* was demonstrated in upregulation of the proliferation and migration of vascular SMCs [26]. Moreover, in vivo molecular/genetic loss-of-function approaches indicate an important function of CaMKII*δ* in promoting injury-induced vascular wall remodeling [27, 28], flow dependent remodeling [29], and angiotensin II-induced vascular wall hypertrophy. Expression of endogenous CaMKII*γ* in VSM after vascular injury is permissive of coupling CaMKII*δ*-enriched holoenzymes to promotion of VSM synthetic phenotype functions [14].

CaMKII has been implicated as a regulator of smooth muscle contraction for more than a decade [11–13, 30, 31]. Early reports indicated that *γ* isoform of CaMKII was highly expressed in differentiated smooth muscle cells that acquired contractile activity [9, 12]. However, whether CaMKII*γ* plays a role in differentiation of mesenchymal stem cells into contractile smooth muscle cells remains unclear. To our knowledge, this study is the first to demonstrate that CaMKII*γ* isoforms are involved in modulation of smooth muscle cell differentiation of adult mesenchymal stem cells.

Firstly, according to our previous study [5], we treated hASCs with TGF-*β*1 and BMP4 in combination and observed smooth muscle contractile proteins were highly expressed with induction, indicating a successful differentiation to SMCs. The highly expressed smooth muscle contractile proteins were CArG-dependent smooth muscle differentiation markers, because TGF-*β*1 and BMP4 induce CArG-dependent smooth muscle differentiation. We observed a paralleled upregulation of CaMKII*γ*, but not other isoforms of CaMKII, during smooth muscle differentiation of hASCs. To examine the role of CaMKII*γ* in the control of contractile gene expression, we overexpressed CaMKII*γ* in the hASCs; this overexpression caused upregulation of smooth muscle contractile markers. We also used siRNA to knockdown CaMKII*γ* expression. We found that inhibiting CaMKII*γ* resulted in suppression of smooth muscle contractile relevant proteins expression as determined by western blot analysis and immunofluorescent staining. These results indicated that, among the four isoforms of the CaMKII, only CaMKII*γ* promotes smooth muscle differentiation of hASCs. Saddouk et al. [14] indicated that decrease of CaMKII*γ* coincided with decrease of contractile smooth muscle phenotype markers in the medial wall of carotid arteries. These results are also consistent with Kim et al.'s reports [12], in which they treated aorta tissue with non-variant-specific-CaMKII*γ* antisense oligonucleotides and found that contractility of aorta was decreased significantly when it was stimulated with KCI. Taken together, these results including ours indicate that CaMKII*γ* is one of the fundamental players that improves differentiation of hASCs into contractile smooth muscle phenotype. In addition, CaMKII*γ* participates in CArG-dependent smooth muscle differentiation.

In conclusion, results of this study demonstrated that *γ* isoform of CaMKII is upregulated in the differentiation of hASCs into smooth muscle cells. Interfering expression of CaMKII*γ* with siRNA significantly decreased contractile proteins expression. Further work needs to be done to explore molecular mechanism responsible for regulatory effect of CaMKII*γ* in differentiation of contractile SMC phenotype.

## Figures and Tables

**Table 1:** qRT-PCR primer sequences.

Name	Sequence (5′ → 3′)	
a-SMA	Forward	GGTGATGGTGGGAATGGG
	Reverse	GCAGGGTGGGATGCTCTT

Calponin	Forward	ATGTCCTCTGCTCACTTCA
	Reverse	TTTCCGCTCCTGCTTCTCT

SM22a	Forward	AACAGCCTGTACCCTGATGG
	Reverse	CGGTAGTGCCCATCATTCTT

SM-MHC	Forward	TGCTTTCGCTCGTCTTCC
	Reverse	CGGCAACTCGTGTCCAAC

Smoothelin 2	Forward	CCCCTGAGATTGCCCAAAACT
	Reverse	CATGGGTGATAGAGCCGCAG

ACLP	Forward	ACCCACACTGGACTACAATGA
	Reverse	GTTGGGGATCACGTAACCATC

CaMKII*α*	Forward	ACCACTACCTGATCTTCGACC
	Reverse	CCGCCTCACTGTAATACTCCC

CaMKII*β*	Forward	GCACACCAGGCTACCTGTC
	Reverse	GGACGGGAAGTCATAGGCA

CaMKII*γ*	Forward	GTCTGTCAACGATCCACGGT
	Reverse	TCTGCCTGCCAACTGAGAAG

CaMKII*δ*	Forward	AGGGCTTTCACTACTTGGTGT
	Reverse	AGCCAAAGTCTGCCAATTTCA
